# *Streptomyces* Diversity Maps Reveal Distinct High-Specificity Biogeographical and Environmental Patterns Compared to the Overall Bacterial Diversity

**DOI:** 10.3390/life14010011

**Published:** 2023-12-20

**Authors:** Nuttapon Pombubpa, Chayaporn Lakmuang, Pornnapat Tiwong, Chompoonik Kanchanabanca

**Affiliations:** 1 Department of Microbiology, Faculty of Science, Chulalongkorn University, Bangkok 10330, Thailand; nuttapon.po@chula.ac.th (N.P.); 6480177920@student.chula.ac.th (C.L.); pornnapat.tiwong@gmail.com (P.T.); 2 Microbiome Research Unit for Probiotics in Food and Cosmetics, Chulalongkorn University, Bangkok 10330, Thailand

**Keywords:** *Streptomyces*, microbial biogeography, *Streptomyces* biodiversity, *Streptomyces* diversity map, microbiomics

## Abstract

Despite their enormous impact on the environment and humans, the distribution and variety of the biggest natural secondary metabolite producers, the genus *Streptomyces*, have not been adequately investigated. We developed representative maps from public EMP 16S rRNA amplicon sequences microbiomics data. *Streptomyces* ASVs were extracted from the EMP overall bacterial community, demonstrating *Streptomyces* diversity and identifying crucial diversity patterns. Our findings revealed that while the EMP primarily distinguished bacterial communities as host-associated or free-living (EMPO level 1), the *Streptomyces* community showed no significant difference but exhibited distinctions between categories in EMPO level 2 (animal, plant, non-saline, and saline). Multiple linear regression analysis demonstrated that pH, temperature, and salinity significantly predicted *Streptomyces* richness, with richness decreasing as these factors increased. However, latitude and longitude do not predict *Streptomyces* richness. Our *Streptomyces* maps revealed that additional samplings in Africa and Southeast Asia are needed. Additionally, our findings indicated that a greater number of samples did not always result in greater *Streptomyces* richness; future surveys may not necessitate extensive sampling from a single location. Broader sampling, rather than local/regional sampling, may be more critical in answering microbial biogeograph questions. Lastly, using 16S rRNA gene sequencing data has some limitations, which should be interpreted cautiously.

## 1. Introduction

The genus *Streptomyces* is classified as a group of Gram-positive filamentous bacteria [1,2]. The distinct life cycle of *Streptomyces* includes the onset of vegetative mycelia autolysis via a programmed cell death-like mechanism to provide nutrients required for the development of aerial mycelia, which then develop into a long chain of spores. Meanwhile, to protect such nutrients from competing microorganisms, *Streptomyces* species produce bioactive small molecules, which humans have used in the pharmaceutical industry, making the genus well-known as a major antibiotic producer [3,4,5,6,7]. This group can be found in various environments, especially soil and sediments [1,8]. *Streptomyces* species are of interest because of their ability to produce essential bioactive secondary metabolites such as antibiotics. It has been indicated that *Streptomyces* antibiotic biosynthesis pathways account for about 5–10% of their genome [9,10]. Moreover, previous research has also shown that *Streptomyces* strains are crucial not only to human health but also to the agricultural system and global ecosystem. As of now, more than 600 species are known to be included in the genus [11]. Members in the genus *Streptomyces* are abundant in the soil as they play a crucial role in the cycle of carbon trapped in insoluble organic debris, especially from plants and fungi, by producing several hydrolytic exoenzymes for macromolecule digestion [3]. Many researchers have investigated *Streptomyces* spp. as a rhizosphere microbe and endophyte, which has showed that these *Streptomyces* produce metabolites to prevent plants from other bacterial and fungal infections [12,13,14,15]. In recent decades, the isolation and exploitation of *Streptomyces* spp. from conventional environments, such as soil and seawater, for the discovery of novel compounds have resulted in the rediscovery of previously known compounds. Therefore, the recent isolation of *Streptomyces* has been directed towards exotic and unexplored sources, including hypersaline marine environments, marine sediment, volcanic areas, hyper-arid deserts and cryoenvironments, and those associated with plants and animals [5,16,17,18,19,20]. Despite their crucial contribution to the environment and humans, curated documentation about large-scale *Streptomyces* distribution and diversity is very limited to local and regional investigation, which needs to be investigated further using a standardized procedure on a global level.

Previous studies of *Streptomyces* spp. are based on cultivation, isolation, and first-generation sequencing, such as the Sanger sequencing method, which showed *Streptomyces* spp. regional biography [21,22]. However, there are limitations to using traditional methods in many ecosystems. Culturing procedures often underestimate the true microbial diversity and are sometimes unreliable for microbial community characterization [23]. Additionally, many potential factors contribute to global microbial biogeography, such as environments, geography, pH, temperature, salinity, etc. [24,25,26]. As a result, a representative *Streptomyces* global distribution profile is difficult to decipher using traditional cultivation and isolation alone. Moreover, it is very complicated to compare or link between studies when different methods are used to assess *Streptomyces* diversity. With technological advancement, we can now rapidly obtain this information from all around the world using next-generation sequencing (NGS). The NGS method can obtain a higher number of sequences and provide more details regarding microbial communities. This method was used in the Earth Microbiome Project (EMP) [27,28,29,30,31], which will not only provide an opportunity for a standardized procedure (sequencing platform, amplicon region, and amplicon metabarcoding protocol) on a global level of bacterial diversity, including *Streptomyces* spp., but also eliminates the complication and complexity in diversity comparison using public sequences from many projects that used different procedures to acquire the sequences.

Similar to the EMP, other platforms for curated environment microbiome databases and resources have emerged for extreme environment microorganisms, a global catalog of the urban microbiome, northern Chile’s arid and desert microbiome, and the soil microbiome. These platforms include the Extreme Microbiome Project (XMP) [32], the International Metagenomics and Metadesign of Subways and Urban Biomes (MetaSUB) Consortium [33], the Atacama Database [34], and Biomes of Australian Soil Environments (BASE) [35], respectively. Nevertheless, the EMP is a massive crowd-sourced database comprising around 100 studies, which makes it the most suitable source for our study. The EMP studied global microbial diversity using NGS [31]. As a baseline documentation for a general global bacterial diversity pattern, the EMP showed that the aspect of host association was crucial for the overall bacterial communities [31]. The overall bacterial richness was lower in host-associated communities than in free-living populations (except plant rhizospheres in EMP, which are similar to free-living soil communities) [31]. In free-living samples, bacterial compositions in saline samples are distinct from non-saline samples. In non-host-associated habitats, the alpha diversity analysis of the large EMP dataset demonstrates a small but significant trend toward increasing species richness at lower latitudes. While the EMP is the beginning of deciphering bacterial global biogeography, the authors also indicated that the rate at which data are generated outpaces our ability to extract useful information. According to the distinct morphology of the genus *Streptomyces*, e.g., tip extension mycelia and long-lived spores and the ability to produce antagonistic compounds against microorganisms, altogether, it is worth investigating whether the alpha and beta diversity of *Streptomyces* spp. is similar to the overall bacterial communities presented in the EMP or not. To unravel the large, complicated EMP dataset at a finer resolution, *Streptomyces* ASVs diversity data will be obtained from the EMP bacterial dataset to investigate alpha and beta diversity in comparison to the EMP diversity results and to develop baseline documentation for future *Streptomyces* diversity studies.

Therefore, the main objectives of this study are to determine whether the *Streptomyces* composition pattern is similar or different from the overall microbial community and to establish comprehensive *Streptomyces* diversity maps. By leveraging the utilization of the EMP 16S rRNA gene (16S) dataset and focusing on *Streptomyces* diversity specifically, we predict that (1) the *Streptomyces* ASVs alpha and beta diversity pattern is different from the overall global microbial community, in which different geographical locations and environments structure the *Streptomyces* communities with respect to both richness and composition, and that (2) increasing the number of sample collections in a particular location did not always result in greater *Streptomyces* ASVs richness, though the 16S rRNA gene may not have sufficient resolution to differentiate all *Streptomyces* species. Additionally, given the data we retrieved from the public database, more sampling is needed in many locations to capture the global diversity of *Streptomyces*. Insights from this work will not only help determine and document what we know about *Streptomyces* diversity using a reliable, standardized procedure, but will also provide potential indications of the future sampling and surveys that should be investigated to understand the true global *Streptomyces* diversity. Additionally, biogeography data on *Streptomyces* diversity would help pinpoint the locations for *Streptomyces* isolation, perhaps leading to a potential novel strain and drug discoveries.

## 2. Materials and Methods

### 2.1. Data Retrieval

Sample processing, sequencing, and core amplicon data analyses were performed as part of the Earth Microbiome Project (www.earthmicrobiome.org (accessed on 9 March 2022)), and all amplicon sequence data and metadata have been made public through the EMP data portal (https://qiita.ucsd.edu/emp/ (accessed on 9 March 2022)). The 16S rRNA amplicon sequences data were retrieved from the EMP Zenodo archive (https://doi.org/10.5281/zenodo.890000 (accessed on 9 March 2022)) [31]. Briefly, for all these samples, DNA extraction and 16S rRNA amplicon sequencing were performed following the standard protocols outlined in the EMP (http://www.earthmicrobiome.org/protocols-and-standards/16s (accessed on 9 March 2022)). The sequence data underwent demultiplexing and minimal quality filtering through the QIIME 1.9.1 script, split_libraries_fastq.py60, utilizing a Phred quality threshold of 3 and default parameters to produce study-specific FASTA sequence files. Subsequently, the sequence data underwent error filtering and trimming to the length of the shortest sequencing run (90 bp) using the Deblur software 1.0.0. After retrieving the data, amplicon sequence variants (ASVs), specifically within the *Streptomyces* genus, were extracted from the EMP data. The Illumina amplicons used for this study targeted the V4 region of the 16S rRNA gene (rDNA), with a sequence length of ~390 base pairs. “emp_cr_silva_16S_123.release1.biom” was used as a source for an amplicon sequence variants (ASVs) table. This biom file also provided taxonomy data referencing the SILVA high-quality ribosomal RNA database [36]. “emp_qiime_mapping_release1_20170912.tsv” was used as a mapping file for data analysis. The raw ASVs table (307,572 ASVs) and taxonomy from the EMP 16S rRNA amplicon sequences data consisted of 27,406 samples collected worldwide.

### 2.2. Bioinformatics and Data Processing

This research focused on representative *Streptomyces* spp. diversity from 43 locations and 96 studies within the EMP data; therefore, 307,572 raw ASVs in an ASV table and taxonomy were filtered to obtain only *Streptomyces* ASVs using the “filter_taxa_from_otu_table.py” function in Qiime [37]. Then, the biom table containing only *Streptomyces* ASVs was converted to an ASV table with taxonomy information for each associated ASV using the “biom_convert” function [38]. After data processing, there were 485 ASVs at the ranks of species and subspecies of *Streptomyces* taxa from 34 countries (the samples summary table is available at https://github.com/natpombubpa-lab/Strep_biogeo/blob/main/Sample_metadata_summary.csv). Singleton ASVs were removed resulting in 429 ASVs, by using “prune_taxa” and “taxa_sums” from the Phyloseq packages [39] in R version 4.0.2 [40] and R studio version 1.3.1093 [41].

### 2.3. Data Analysis

*Streptomyces* alpha diversity results from the rarefied ASVs data were compared to (1) overall bacteria alpha diversity using the same analysis pipeline and (2) directly compared to Thompson et al., 2017 overall bacteria alpha diversity results for confirmation. The Phyloseq package was used to assess filtered microbial ASV alpha diversity, beta diversity, and taxonomic composition. Levene’s test, Bartlett’s test, and Hartley’s Fmax test were used in R to check for homoscedasticity in alpha diversity data variances [42]. The ‘Anova’ function in R was used to evaluate homoscedasticity data (alpha diversity), and the ‘TukeyHSD’ function was used to conduct pairwise multiple comparisons (Tukey test). A type = ‘III’ ANOVA was used to adjust for the unbalanced design comparison. Multiple linear regression analysis on environmental factors that are available for the majority of the samples (pH, temperature, and salinity) were performed using the ‘lm’ function for both *Streptomyces* and overall bacteria. To compare beta diversity amongst the samples, PERMANOVA was employed with the ‘adonis’ function in the ‘vegan’ package and visualized using PcoA in R using Bray–Curtis distance [43]. Diversity analysis was performed using the Earth Microbiome Project Ontology (EMPO), including EMPO level 1–3. EMPO level 1 includes free-living and host-associated. EMPO level 2 includes saline, non-saline, animal, and plant. EMPO level 3 includes water (saline), sediment (saline), hypersaline (saline), surface (saline), water (non-saline), sediment (non-saline), soil (non-saline), surface (non-saline), aerosol (non-saline), animal distal gut, animal proximal gut, animal secretion, animal surface, animal corpus, plant surface, plant rhizosphere, and plant corpus. Then, *Streptomyces* beta diversity results were directly compared to overall bacteria beta diversity results in Thompson et al., 2017 [31]. Bioinformatics, data processing, and data analysis coding scripts are available on GitHub at the following link: https://github.com/natpombubpa-lab/Strep_biogeo.

## 3. Results

### 3.1. Does the Environment Shape Streptomyces Composition and Richness Differently Compared to the Overall Bacterial Community Pattern Found in the EMP?

**Figure 1:**
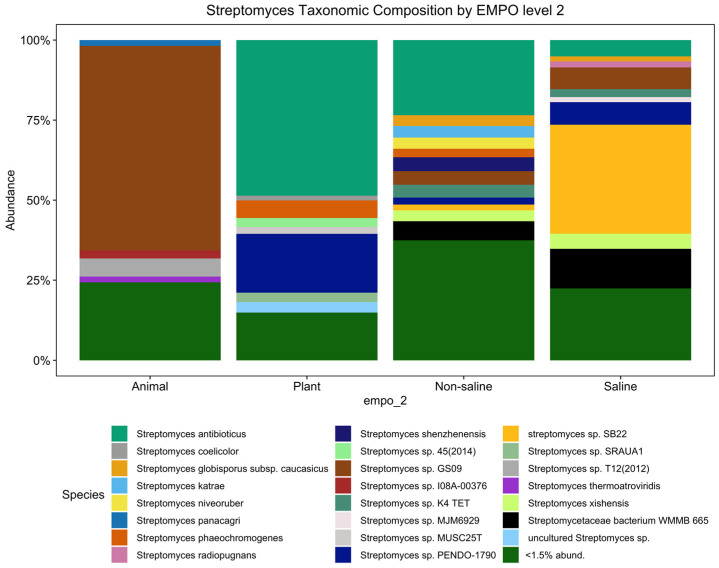
*Streptomyces* taxonomic composition bar plot showing distinct composition among animal, plant, non-saline, and saline samples (<1.5% abund. = <1.5% relative abundance). Among 429 ASVs, there were 23 *Streptomyces* spp. (27 ASVs) with a relative abundance exceeding 1.5 percent. However, the minor but diverse ASVs (<1.5%) represented 402 taxa (species/subspecies).

**Figure 2:**
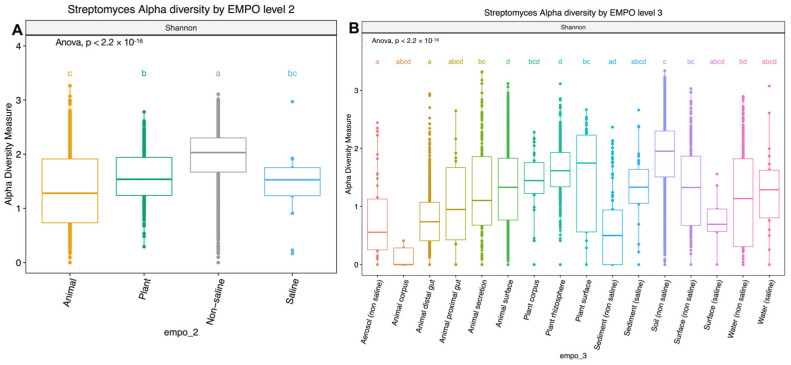
Boxplots showing alpha diversity (Shannon index) comparison of *Streptomyces* spp. in (**A**) EMPO level 2: animal, plant, non-saline, and saline samples and (**B**) EMPO level 3: aerosol (non-saline), animal corpus, animal distal gut, animal proximal gut, animal secretion, animal surface, plant corpus, plant rhizosphere, plant surface, sediment (non-saline), sediment (saline), soil (non-saline), surface (non-saline), surface (saline), water (non-saline), and water (saline). Boxplots show the 25th and 75th percentiles, while the median is shown as lines inside boxes. Error bars show the 1st and 99th percentile. Tukey HSD significant differences (*p* < 0.05) are indicated by different letters.

Although the significant distinction of the bacterial microbial composition in the EMP was host-associated vs. free-living samples [31] (Figure S1A) (ANOVA, F(3,7356) = 387.8, *p* < 2.2 × 10^−16^), the *Streptomyces* community showed no significant difference among host-associated vs. free-living samples. However, the *Streptomyces* community showed distinct patterns between animal and plant samples (Figure 1), which is supported with alpha diversity analysis showing that richness is significantly different (ANOVA, F(3,3211) = 210.4, *p* < 2.2 × 10^−16^) (Figure 2A). However, the *Streptomyces* taxa with the greatest relative abundance of *Streptomyces* spp. are similar between non-saline and saline samples, but both the number and composition of abundant ASVs/strains were distinct among four EMPO groups. In general, *S. antibioticus* was the most common and abundant strain, which was found in all EMPO level 2 categories except in animal samples.

**Figure 3:**
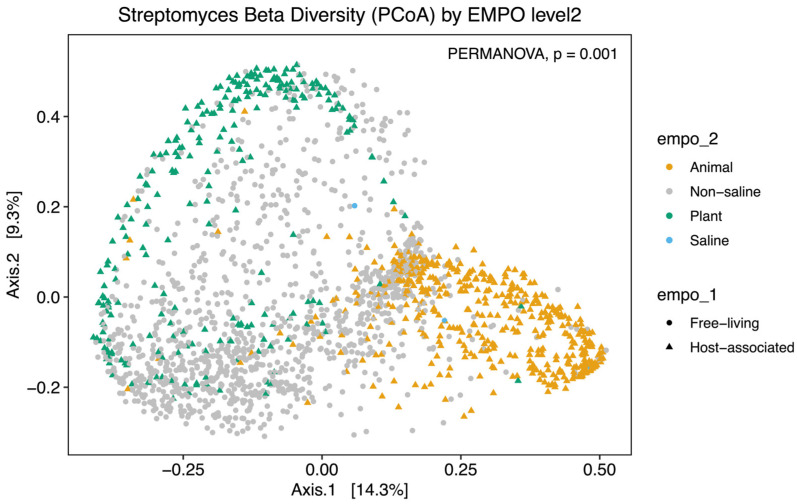
Beta diversity analysis of *Streptomyces* communities. Dissimilarity of *Streptomyces* community composition in the comparison between EMPO levels using PcoA. Different colors indicate EMPO level 2, including the yellow color for animal samples, the light gray color for non-saline samples, the green color for plant samples, and the blue color for saline samples. Circle points show free-living samples, while triangle points indicate host-associated samples. Significant differences among EMPO level 2 (PERMANOVA; *p* < 0.05) were shown on PcoA plots.

Alpha diversity analysis showed significant environmental differences for *Streptomyces* richness for both EMPO level 2 (ANOVA, F(3,3211) = 210.4, *p* < 2.2 × 10^−16^)(Figure 2A) and EMPO level 3 (ANOVA, F(15,3199) = 74.283, *p* < 2.2 × 10^−16^, Figure 2B), similar to the overall bacteria richness analysis in our study (Figure S1B,C) and Thomspon et al., 2017 [31]. In the EMPO level 2 alpha diversity comparison, *Streptomyces* richness was significantly higher in non-saline samples, whereas *Streptomyces* richness in animal and saline samples was not significantly different (Figure 2A). While the overall microbial richness was lower in host-associated (animal and plant) samples than in free-living (non-saline and saline) samples, from the EMP report and our analysis (Figure S1A), specifically focusing on *Streptomyces* spp. richness alone showed that *Streptomyces* richness differences were not significantly shaped by EMPO level 1 (host vs. free-living) but were determined by EMPO level 2 (animal, plant, non-saline, and saline). Additionally, *Streptomyces* richness at the ASV/strain level was also shaped differently by EMPO level 3. *Streptomyces* richness was highest in soil (non-saline) samples and lowest in sediment (non-saline) samples (Figure 2B). We also investigated how EMPO shaped beta diversity among the *Streptomyces* community. Beta diversity analysis showed significant differences among samples based on EMPO (PERMANOVA, *p* = 0.001) (Figure 3). The differences in *Streptomyces* beta diversity were visualized in a principal coordinate analysis (PcoA) plot (Figure 3). Microbial communities were shown to be tightly grouped by EMPO level 2 for animal samples in the PcoA plot. Plant and non-saline samples were not clearly separated from each other. EMPO level 1 alone (free-living and host-associated) also did not shape the *Streptomyces* community pattern.

Additionally, multiple linear regression analysis on environmental factors, which are available for the majority of the samples (pH, temperature, and salinity), showed that pH, temperature, and salinity significantly predict *Streptomyces* richness (*p* < 0.05) in which richness decreases as pH, temperature, and salinity increase. In contrast, only pH and salinity significantly predict overall bacteria richness (*p* < 0.05). In our map, *Streptomyces* spp. richness was higher near the equator and lower near the north and south poles. However, linear regression analysis showed neither latitude nor longitude predicts *Streptomyces* richness (*p* > 0.05).

### 3.2. A Comprehensive Streptomyces Diversity Map: Do Geographical Locations Structure Streptomyces Community Diversity?

**Figure 4:**
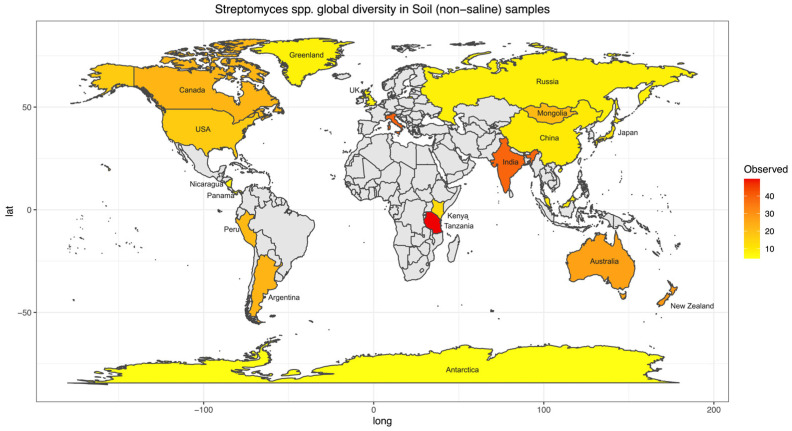
*Streptomyces* diversity map in soil (non-saline) samples showing species richness distribution worldwide. The average number of observed ASVs was shown as a gradient from lowest (yellow) to highest (red) diversity while grey color showed no *Streptomyces* or sample. Twenty-one regions were included in the soil (non-saline) *Streptomyces* diversity analysis. Note, some regions were not indicated on the map because of space limitations.

**Figure 5:**
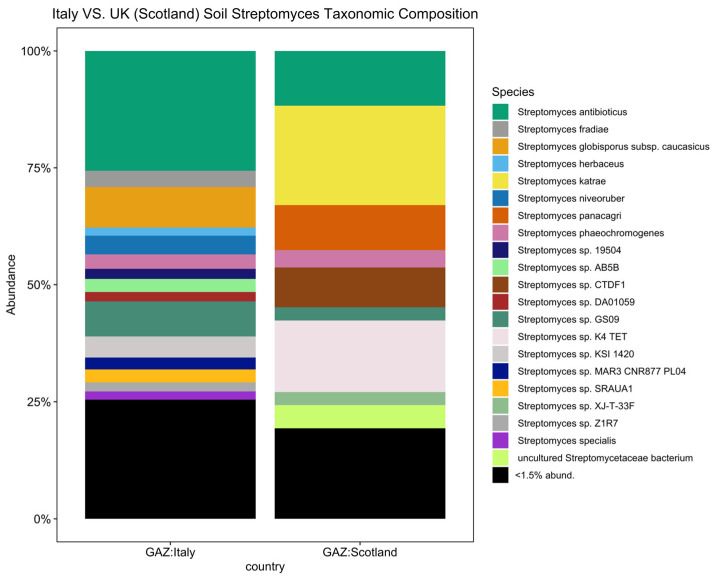
*Streptomyces* taxonomic composition bar plot showing distinct composition between Italy and the United Kingdom (Scotland) (<1.5% abund. = <1.5% relative abundance). In total, there were 21 *Streptomyces* spp. with a relative abundance greater than 1.5 percent.

To further investigate biogeographical and environmental specificity, thirteen *Streptomyces* diversity maps were established based on the number of observed ASVs for each EMPO level 3, including (1) soil (non-saline) (Figure 4), (2) water (non-saline), (3) sediment (non-saline), (4) surface (non-saline), (5) aerosol (non-saline), (6) sediment (saline), (7) surface (saline), (8) water (saline), (9) animal surface, (10) animal secretion, (11) animal proximal gut, (12) animal distal gut, and (13) plant rhizosphere (Supplementary Information (SI), Figures S2–S13). The *Streptomyces* diversity map in soil (non-saline) samples revealed significant differences in *Streptomyces* richness across geographical locations (ANOVA, F(20,3480) = 125.6, *p* < 2.2 × 10^−16^, Figure 4). The number of observed soil *Streptomyces* ASVs were greatest in Tanzania and the second highest level of richness was observed in Italy. In contrast, low soil *Streptomyces* ASV richness was found in Antarctica, China, Greenland, Japan, Kenya, Malaysia, Nicaragua, Panama, Russia, and the United Kingdom (Scotland). While the number of soil samples in Italy (48 samples) was lower than in the United Kingdom (Scotland) (453 samples), the *Streptomyces* community was not only different in richness (highest richness in Italy though lower number of samples) but also distinct in ASV/strain composition (Figure 5). To confirm this pattern, further analysis using simple linear regression was performed to test if sample numbers significantly predicted *Streptomyces* richness. It was found that sample numbers do not significantly predict *Streptomyces* richness (*p* > 0.05).

By mapping *Streptomyces* richness, general biogeographical patterns were observed for each EMPO level 3 environmental category. Generally, in non-saline (water, surface, and aerosol) samples, *Streptomyces* richness was high in the USA (Figures S2–S4). However, no water sample was taken from Asia, Africa, and Oceania. In contrast, in non-saline (sediment) samples, *Streptomyces* richness was high in Brazil but low in the USA and China (Figure S5). In saline (sediment and surface) samples, *Streptomyces* richness was low in the USA (Figures S6 and S7). However, in water (saline) samples, *Streptomyces* richness was high in Mexico but low in Brazil and Norway (Figure S8). In animal surface and proximal gut samples, *Streptomyces* richness was low in the USA (Figures S9 and S10). In animal secretion samples, *Streptomyces* richness was high in the USA and low in Venezuela (Figure S11). In animal distal gut samples, *Streptomyces* richness was generally high in South America and low in Germany and Australia (Figure S12). Lastly, in plant rhizosphere samples, *Streptomyces* richness was high in the USA and low in Japan (Figure S13).

## 4. Discussion

Our key results indicated that (1) while our *Streptomyces* maps revealed that samples were collected from all over the world, additional samplings, particularly in Africa and Southeast Asia, are still needed. Moreover, increasing the number of sample collections in a given location did not necessarily result in increasing *Streptomyces* diversity, and (2) the *Streptomyces* community showed no significant difference but exhibited distinctions between categories in EMPO level 2, whereas the EMP primarily distinguished bacterial communities as host-associated or free-living (EMPO level 1).

Comprehensive Streptomyces diversity maps: Current sampling and future survey

Previously, *Streptomyces* diversity and biogeography were investigated at a regional scale, such as in the USA [21,22] and New Zealand [44]. The investigators used different methods, locations, and environments, especially for a very diverse *Streptomyces* genus. With the high-throughput sequencing method employed in New Zealand, latitude pattern was not the key driver for *Streptomyces* diversity. The approach differed from that in the USA, which used traditional culturing and sequencing techniques, leading to non-comparable results. Thus, the inconsistency in the sample preparation and sequencing methodology was presumably the limitation in interpreting an investigation. Moreover, *Streptomyces* studies oftentimes focused solely on soil collection while other environments were not usually investigated, most likely because they are considered soil/sediment inhabitants. It is believed that one gram of soil is estimated to contain 10^7^ colony-forming units (CFU) of soil *Actinobacteria* [45] in which *Streptomyces* represents 50% of the total population [46]. Our comprehensive *Streptomyces* spp. maps helped fill these gaps and overcome this limitation since the same EMP standard procedure was used for the entire global sample collections in different environments [30]. Similar to other previous studies, soil samples were the most prevalent, and the *Streptomyces* spp. map was the most completed map of all EMPO level 3 categories (Figure 4). According to our map, *Streptomyces* spp. richness was higher near the equator and lower near the north and south poles, which was similar to the latitudinal pattern found earlier in the USA [21,22]; however, this pattern is not statistically significant (*p* > 0.05). While this pattern differed from the New Zealand [44] study, it could possibly explain the limitation of comparing different methods rather than local/regional sampling vs. global collection. At present, soil *Streptomyces* collection from the EMP represents a good starting point for biodiversity documentation. Still, our soil *Streptomyces* map showed that many countries, regions, and continents need to be taken into account, including Europe, the Middle East, Southeast Asia, and especially Africa. Although the map may show that there was no data from Africa, some soil *Streptomyces* research has actually been conducted, and new *Streptomyces* species have also been found in African soil [47]. However, a standard NGS procedure should be employed and added to the comprehensive soil *Streptomyces* map to investigate and compare the true diversity of *Streptomyces* spp. in African soil to global diversity. As the crucial antibiotics producers [9,10], investigating African soil would be essential to future *Streptomyces* research, especially when our current data from Tanzania showed that *Streptomyces* richness was the highest in the world. As a result, Africa is currently a black box for *Streptomyces* diversity investigation, and it is possible that many countries may have novel and essential *Streptomyces* spp. awaited to be found. Additionally, according to the review of new *Streptomyces* species published between January 2015 and December 2020, among 135 new species, 75 species were isolated from the terrestrial environment (non-saline soil) [48]. Whereas 6 novel species were from Africa, a total of 62 species were isolated from Asia, of which 39 species were reported from China, and the rest were from Southeast Asia, especially Thailand. Thus, additional NGS analyses of *Streptomyces* from Southeast Asia would be crucial to add to the *Streptomyces* biogeography map with high diversity potential.

Our *Streptomyces* spp. maps did not only show a comprehensive soil *Streptomyces* map but also indicated other environments where very limited research had been completed. Besides the *Streptomyces* spp. map in soil, other EMPO level 3 maps were still limited to several countries (Figures S2–S13). *Streptomyces* in water (non-saline), sediment (non-saline), surface (non-saline), aerosol (non-saline), sediment (saline), surface (saline), water (saline), animal surface, animal secretion, animal proximal gut, animal distal gut, and plant rhizosphere will need to be investigated further to be able to create more completed, comprehensive global diversity maps. To overcome this limitation, the same standard NGS procedure should be used for future sample collection and added to the maps we generated in this research. Moreover, future surveys would be crucial in some of these environments. For example, *Streptomyces* in marine environments has been shown to be a potentially important source of bioactive substances, many active secondary metabolites, and antimicrobial compounds [49,50,51]. However, our water (saline) *Streptomyces* map showed that there were only samples from Brazil, Mexico, and Norway (Figure S8), which identified the lack of standardized *Streptomyces* surveys in marine environments worldwide. Interestingly, our results showed that *S. radiopugnans* was the ASV/strain only found in saline environments but was not abundant/found in animal, plant, and non-saline samples (Figure 1). *S. radiopugnans* was identified as a radiation-resistant bacteria [52] and has also been shown to produce novel fibrinolytic protease, which has the potential for thrombosis treatment [53], which suggested that other marine *Streptomyces* spp. with novel abilities are likely waiting to be discovered. Lastly, future surveys may not require extensive sampling from a single location because our data showed that a greater number of samples did not always result in greater *Streptomyces* richness (Figure 5). In summary, while comprehensive *Streptomyces* diversity maps were generated as a baseline documentation of current samplings, we clearly need additional sample collection for global *Streptomyces* diversity for other EMPO level 3 environments besides soil across as many countries as possible.

EMP dataset at a finer resolution: *Streptomyces* vs. overall bacterial global diversity environmental pattern

According to the EMP project, we are unable to extract relevant information at the rate at which data are generated [31]. The investigation of the EMP dataset at a finer resolution indicated that the overall bacterial diversity pattern might not necessarily be identical to each ASV/strain diversity. Focusing only on *Streptomyces* richness revealed that overall microbial richness was lower in host-associated samples than in free-living samples in the EMP report. *Streptomyces* richness differences were not significantly shaped by host-associated vs. free-living distinctions but rather by EMPO level 2 (animal, plant, non-saline, and saline). Previous research has shown that *Streptomyces* could be a “plant’s best friend” because of their ability to promote plant growth and protect plants from pathogens as a biocontrol agent [8,15]. As we might expect, our results showed that *Streptomyces* richness was high in plant samples, confirming a solid relationship between *Streptomyces* and plants due to disease suppression and plant–microbe coevolution [8,54]. Similar to plant samples, *Streptomyces* richness was also high in non-saline samples (mostly because of soil samples), which supported previous research showing that *Streptomyces* are generally abundant in soil [55,56]. Therefore, using *Streptomyces* as a case study reveals that researchers should use the overall pattern cautiously since the pattern at a finer resolution could potentially be different. However, a similar pattern between overall bacterial diversity and *Streptomyces* diversity was observed when comparing non-saline to saline samples, which was also observed in previous global bacterial diversity studies [24,31]. As a result, both our and previous research indicated that salinity is likely a global regulator of bacterial diversity, resulting in similar patterns for both higher- and finer-level biodiversity. Nevertheless, investigating other bacterial species would provide more insights into overall diversity vs. specific microbial group differences, especially global diversity regulator identification and species-specific regulator identification.

While investigating *Streptomyces* diversity in the EMP dataset showed that additional analyses of public NGS datasets would be helpful, especially at a finer level, using amplicon sequencing data has some limitations that may also apply to other microbial groups. For example, the 16S rRNA gene may not have sufficient resolution to differentiate all *Streptomyces* species. Previous studies revealed that some *Streptomyces* species may show >99% 16S rRNA sequence similarity (identical amplicon sequences) [57,58]; therefore, multilocus sequence analysis (MLSA) and/or whole genome sequencing (WGS) could be used for better resolution [59]. Additionally, discrepancies exist, particularly in the taxonomy databases utilized, where the EMP employed a rDNA-based SILVA database aligning to NCBI with some conflicts [60]. Conflicts also emerge in the comparison with the Genome Taxonomy Database (GTDB), a standardized microbial taxonomy based on genome phylogeny [61]. For example, an instance of conflict involves *Streptomyces cinereus* (classified under the phylum Actinomycetota, previously Actinobacteria), which is designated as *Moraxella cinereus* (belonging to the phylum Pseudomonadota, formerly Proteobacteria) according to the GTDB taxonomy (https://gtdb.ecogenomic.org/genome?gid=GCA_014647715.1 (accessed on 8 December 2023)). Such discrepancies are not uncommon and extend to polymorphism in multi-copied rDNA, exemplified by cases like *Vibrio* [62]. As genome-based taxonomy gains prominence, the study acknowledges the need to consider GTDB and genome-based taxonomies, recognizing the limitations posed by potential inconsistencies in rDNA-based taxonomies. Nevertheless, investigating *Streptomyces* diversity using 16S rRNA amplicon data analysis is still possible, but these data should be interpreted with caution because they may contain taxonomic disagreement. Additionally, the rarefaction curves, illustrating the total ASVs per sample, revealed that numerous samples reached a plateau, while others did not, implying potential coverage inadequacies in some samples (Figure S14A). Nevertheless, the species accumulation plots exhibited an approaching saturation curve, indicating that collecting more samples would likely result in the recovery of only a limited number of additional ASVs (Figure S14B). Lastly, while the EMP provides substantial representative global sampling, it is important to acknowledge that fine-scale biogeographic patterns, environmental heterogeneity, and other additional factors are likely to significantly influence biogeographic patterns. Moreover, performing data analysis beyond EMPO level 3 was limited by data unavailability and incompleteness. Therefore, future studies can overcome this limitation by adding finer resolution levels in data collection with genome-based taxonomy.

## 5. Conclusions

In conclusion, our study case of *Streptomyces* biogeography confirmed that while our scientific community generates a lot of valuable data globally, a second look at these public datasets could provide new insights that we have not seen before in previous investigations. Currently, the soil *Streptomyces* collection from the EMP serves as a helpful starting point for documenting biodiversity. *Streptomyces* alpha diversity analysis displayed distinctions at EMPO level 2, involving animal, plant, non-saline, and saline environments, rather than at EMPO level 1 as seen in the EMP overall bacterial community. Moreover, increasing *Streptomyces* richness can be predicted by decreasing other biotics factors such as pH, temperature, and salinity. As a result, for other specific microbial patterns, the overall microbial pattern should be interpreted carefully because the pattern at a finer resolution might be different, as shown by using *Streptomyces* as a case study. Furthermore, our soil *Streptomyces* map revealed that data from many other locations, particularly in Africa and Southeast Asia, are still needed. Moreover, NGS analysis of marine environments worldwide would be essential for future *Streptomyces* biodiversity investigations. However, using amplicon sequencing data (16S rRNA gene) has some limitations, which should be interpreted cautiously because it may underestimate biodiversity.

## Data Availability

Data supporting this study are openly available from Strep_biogeo repository at https://doi.org/10.5281/zenodo.7807480.

## Supplementary Materials

The following supporting information can be downloaded at: https://www.mdpi.com/article/10.3390/life14010011/s1, Figure S1: Boxplots showing alpha diversity (Shannon index) comparison of overall bacterial community. (A) EMPO level 1: Free-living and Host-associated, (B) EMPO level 2: Animal, plant, non-saline, and saline samples and (C) EMPO level 3: Aerosol (non-saline), animal corpus, animal distal gut, animal proximal gut, animal secretion, animal surface, plant corpus, plant rhizosphere, plant surface, sediment (non-saline), sediment (saline), soil (non-saline), surface (non-saline), surface (saline), water (non-saline), and water (saline). Boxplots show the 25th and 75th percentiles, while the median is shown as lines inside boxes. Error bars show the 1st and 99th percentile. Tukey HSD significant differences (*p* < 0.05) are indicated by different letters; Figure S2: *Streptomyces* diversity map in water (non-saline) samples showing species richness distribution worldwide. The average number of observed ASVs was shown as a gradient from lowest (yellow) to highest (red) diversity; Figure S3: *Streptomyces* diversity map in surface (non-saline) samples showing species richness distribution worldwide. The average number of observed ASVs was shown as a gradient from lowest (yellow) to highest (red) diversity; Figure S4: *Streptomyces* diversity map in aerosol (non-saline) samples showing species richness distribution worldwide. The average number of observed ASVs was shown as a gradient from lowest (yellow) to highest (red) diversity; Figure S5: *Streptomyces* diversity map in sediment (non-saline) samples showing species richness distribution worldwide. The average number of observed ASVs was shown as a gradient from lowest (yellow) to highest (red) diversity; Figure S6: *Streptomyces* diversity map in sediment (saline) samples showing species richness distribution worldwide. The average number of observed ASVs was shown as a gradient from lowest (yellow) to highest (red) diversity; Figure S7: *Streptomyces* diversity map in surface (saline) samples showing species richness distribution worldwide. The average number of observed ASVs was shown as a gradient from lowest (yellow) to highest (red) diversity; Figure S8: *Streptomyces* diversity map in water (saline) samples showing species richness distribution worldwide. The average number of observed ASVs was shown as a gradient from lowest (yellow) to highest (red) diversity; Figure S9: *Streptomyces* diversity map in animal surface samples showing species richness distribution worldwide. The average number of observed ASVs was shown as a gradient from lowest (yellow) to highest (red) diversity; Figure S10: *Streptomyces* diversity map in animal proximal samples showing species richness distribution worldwide. The average number of observed ASVs was shown as a gradient from lowest (yellow) to highest (red) diversity; Figure S11: *Streptomyces* diversity map in animal secretion samples showing species richness distribution worldwide. The average number of observed ASVs was shown as a gradient from lowest (yellow) to highest (red) diversity; Figure S12: *Streptomyces* diversity map in animal distal gut samples showing species richness distribution worldwide. The average number of observed ASVs was shown as a gradient from lowest (yellow) to highest (red) diversity; Figure S13: *Streptomyces* diversity map in plant rhizosphere samples showing species richness distribution worldwide. The average number of observed ASVs was shown as a gradient from lowest (yellow) to highest (red) diversity; Figure S14: (A) Rarefaction curve was generated using the “rarecurve” function in the R library vegan showing frequency of observed ASVs for each sample. (B) Species accumulation curve was generated using “specaccum” function in the R library vegan representing the number of ASVs found at increasing number of sites (locations).
