# Supplementary material for: Streptomyces Diversity Maps Reveal Distinct High-Specificity Biogeographical and Environmental Patterns Compared to the Overall Bacterial Diversity
Source: Life (Basel). 2023 Dec 20;14(1):11. doi: 10.3390/life14010011 (PMC10821021; doi:10.3390/life14010011)
Supplement: Supplementary file 1 [file life-14-00011-s001.zip › life-2714428-supplementary.pdf]

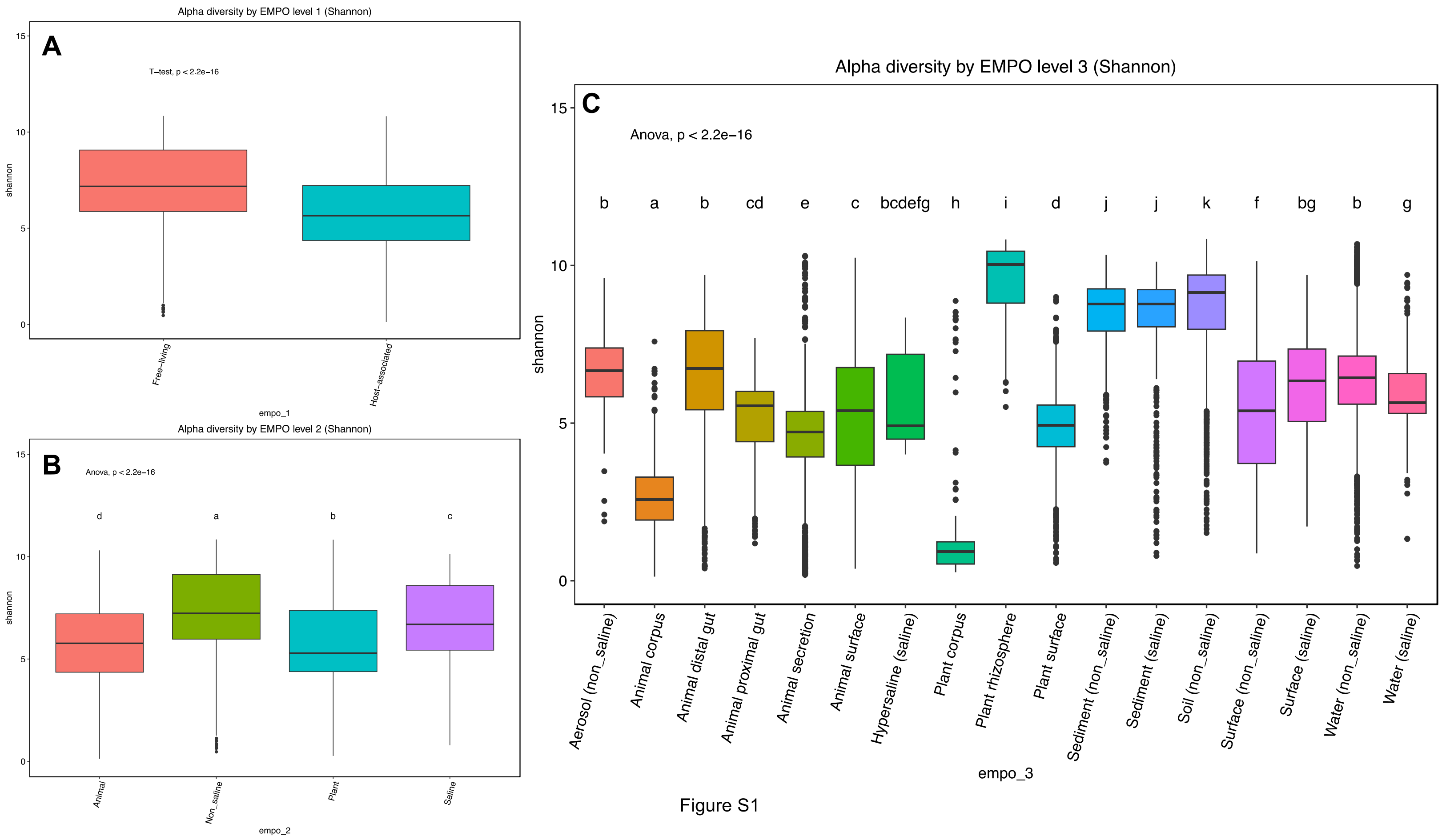

Figure S1. Boxplots showing alpha diversity (Shannon index) comparison of overall bacterial community. (A) EMPO level 1: Free-living and Host-associated, (B) EMPO level 2: animal, plant, non-saline, and saline samples and (C) EMPO level 3: aerosol (non-saline), animal corpus, animal distal gut, animal proximal gut, animal secretion, animal surface, plant corpus, plant rhizosphere, plant surface, sediment (non-saline), sediment (saline), soil (non-saline), surface (non-saline), surface (saline), water (non-saline), and water (saline). Boxplots show the 25th and 75th percentiles, while the median is shown as lines inside boxes. Error bars show the 1st and 99th percentile. Tukey HSD significant differences ($p < 0.05$) are indicated by different letters.

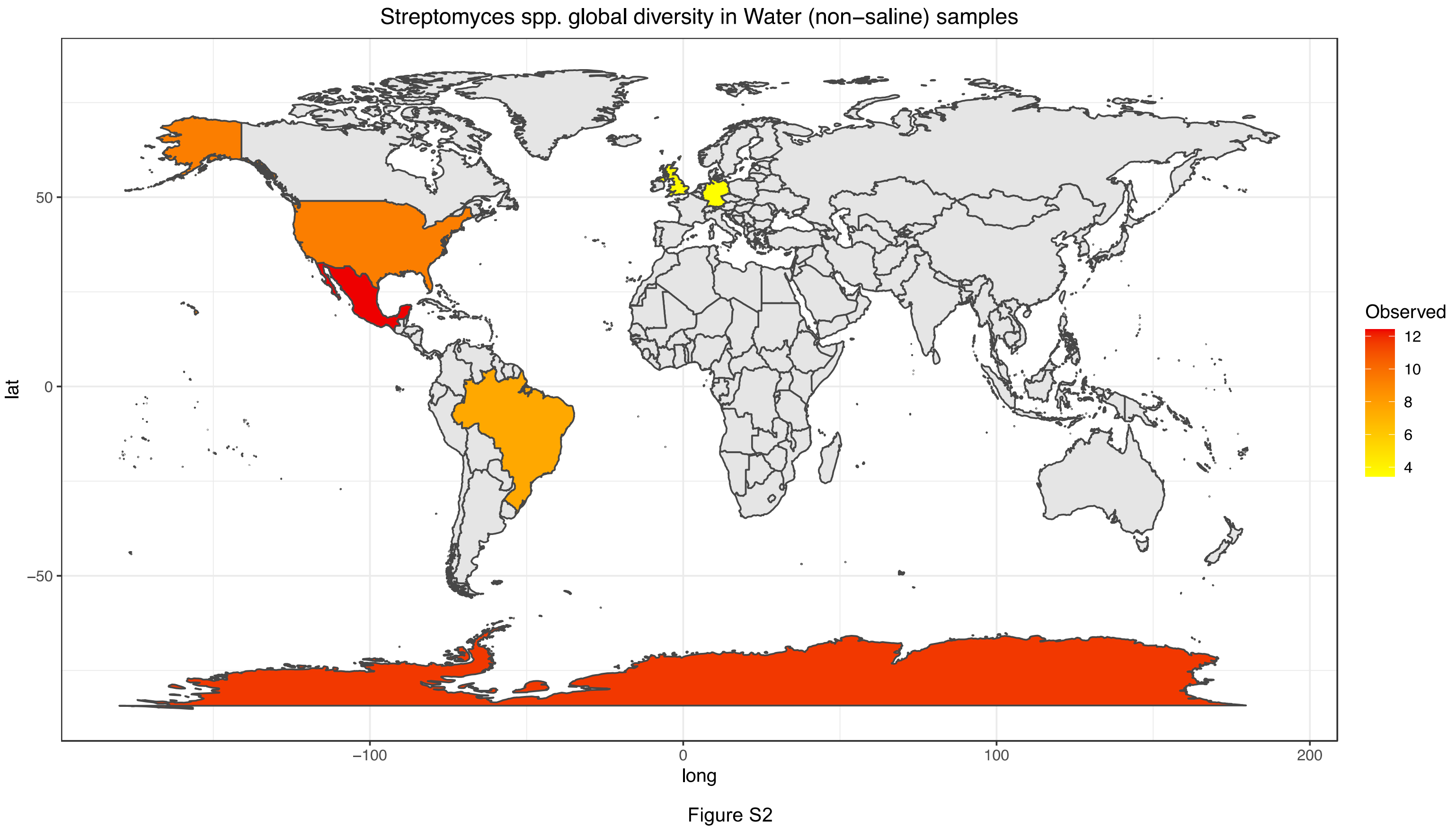

Figure S2. *Streptomyces* diversity map in Water (non-saline) samples showing species richness distribution worldwide. The average number of observed ASVs was shown as a gradient from lowest (yellow) to highest (red) diversity.

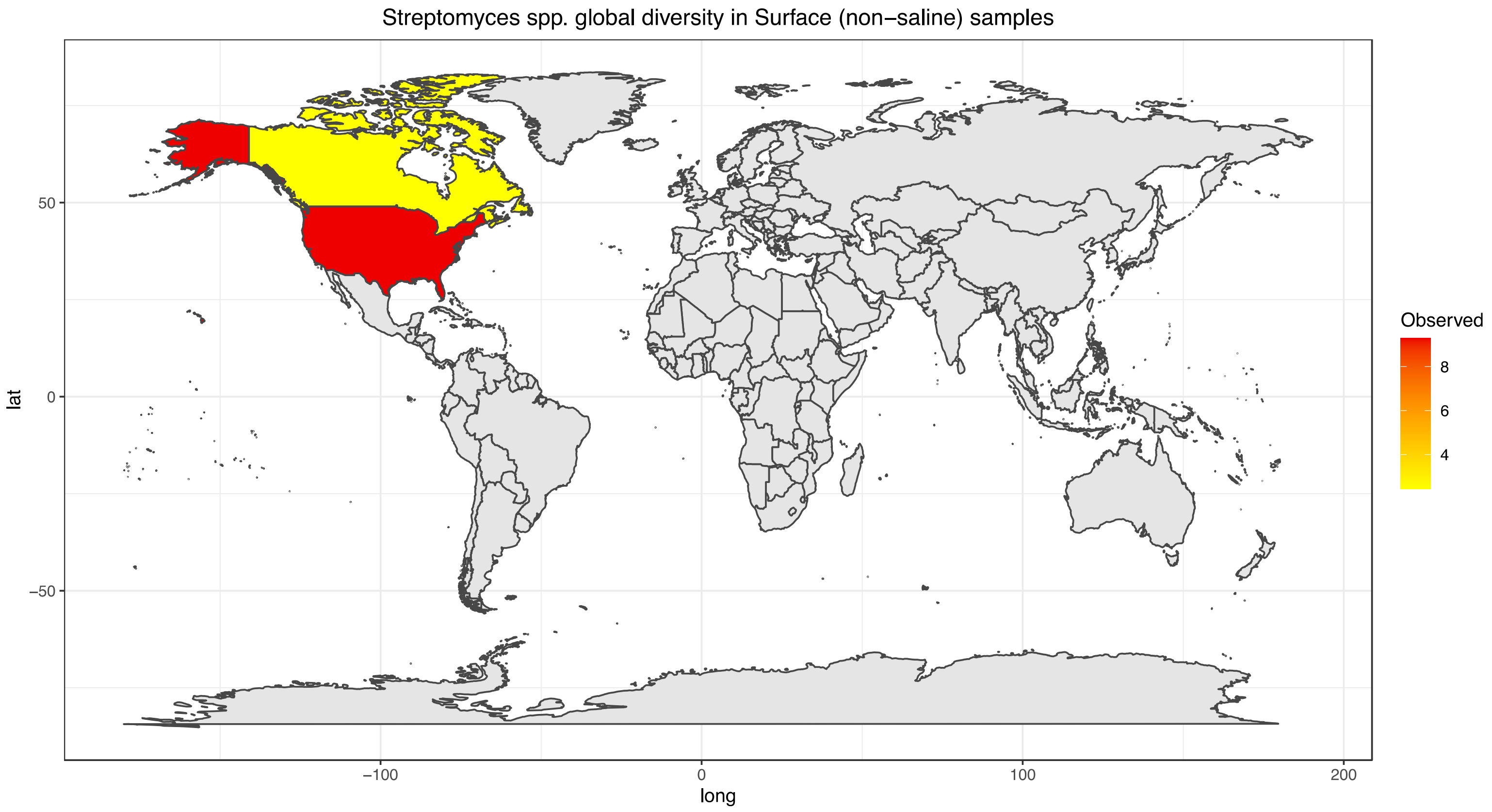

Figure S3

Figure S3. *Streptomyces* diversity map in Surface (non-saline) samples showing species richness distribution worldwide. The average number of observed ASVs was shown as a gradient from lowest (yellow) to highest (red) diversity.

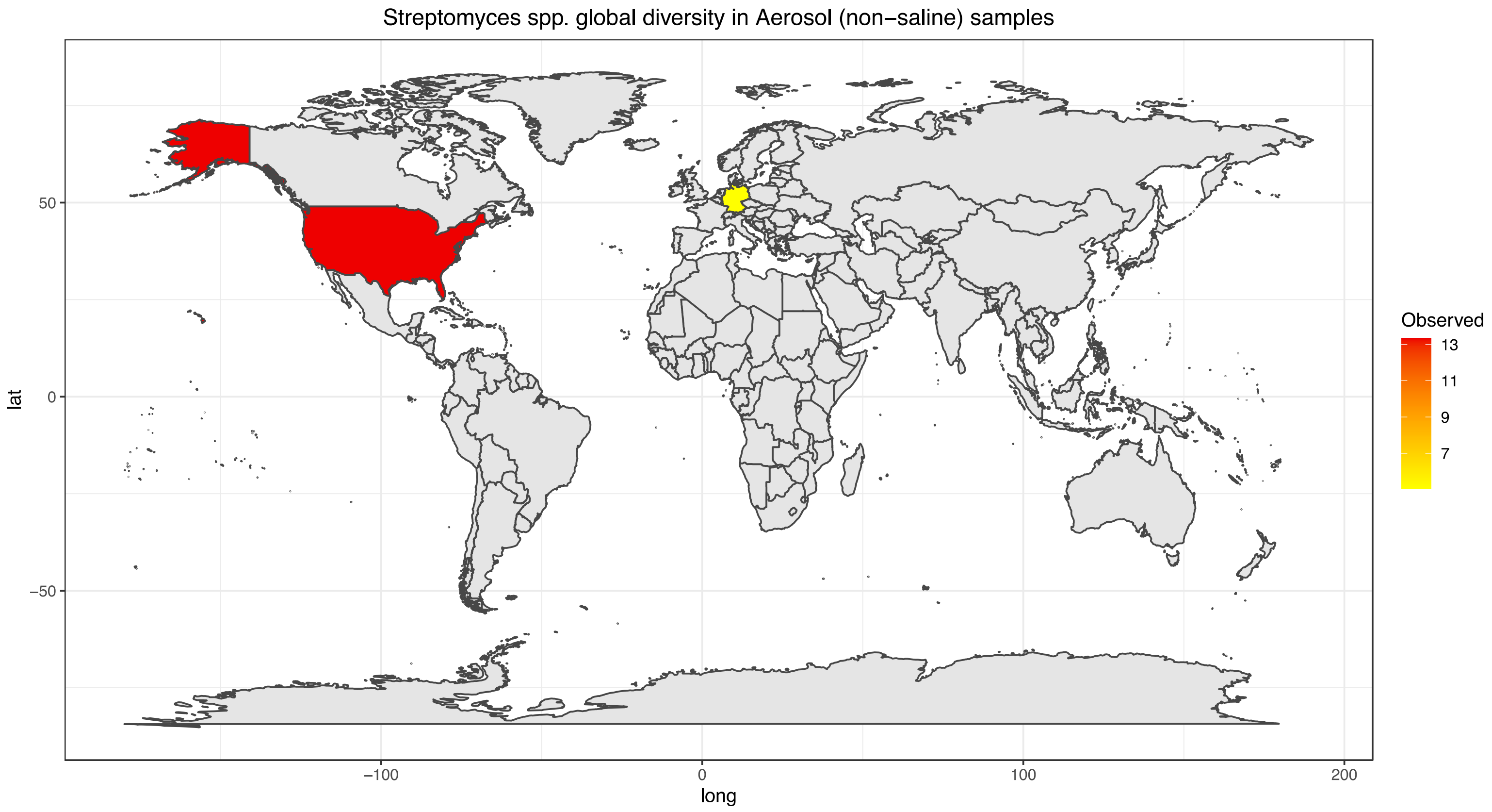

Figure S4

Figure S4. *Streptomyces* diversity map in Aerosol (non-saline) samples showing species richness distribution worldwide. The average number of observed ASVs was shown as a gradient from lowest (yellow) to highest (red) diversity.

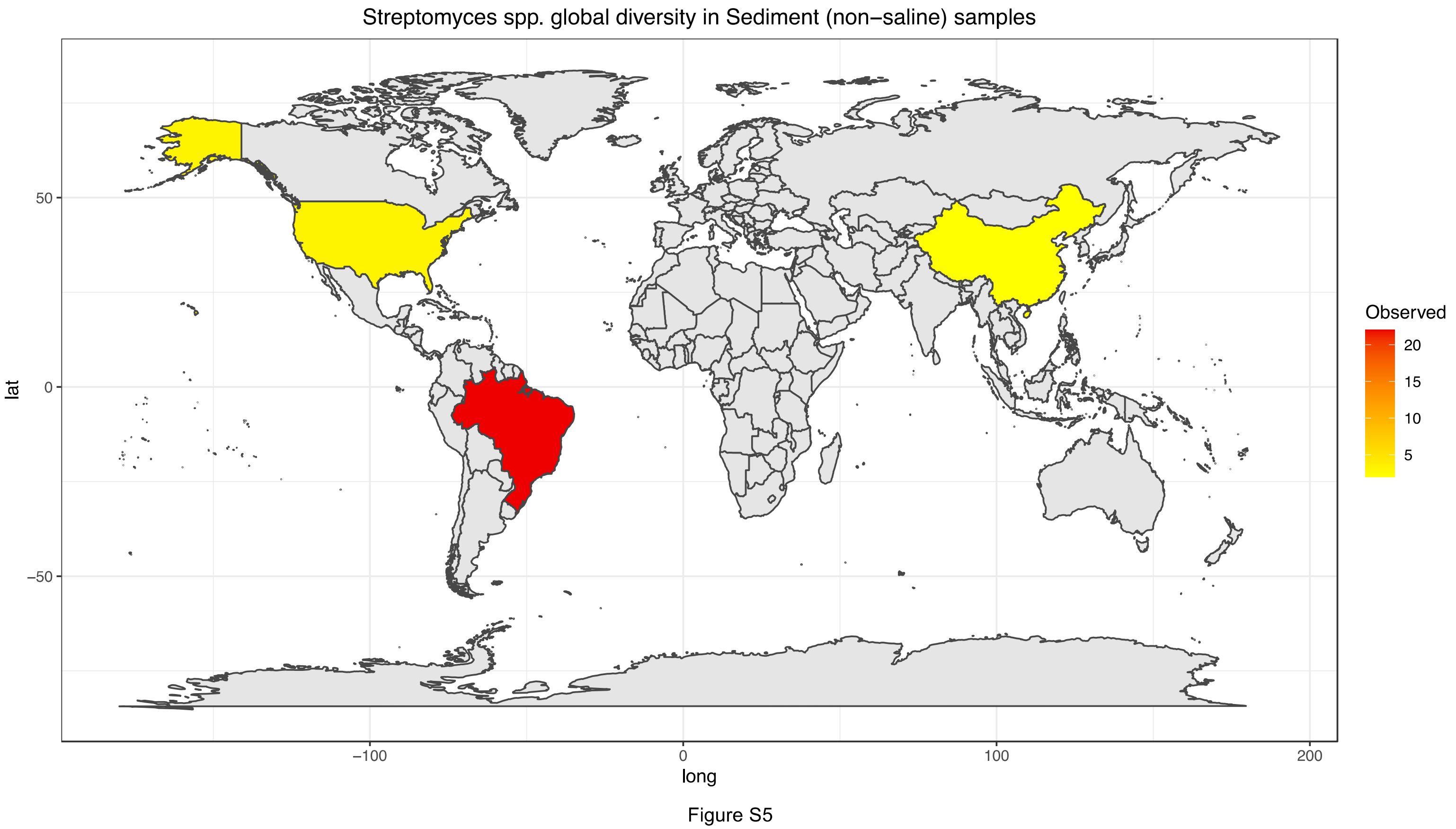

Figure S5. *Streptomyces* diversity map in Sediment (non-saline) samples showing species richness distribution worldwide. The average number of observed ASVs was shown as a gradient from lowest (yellow) to highest (red) diversity.

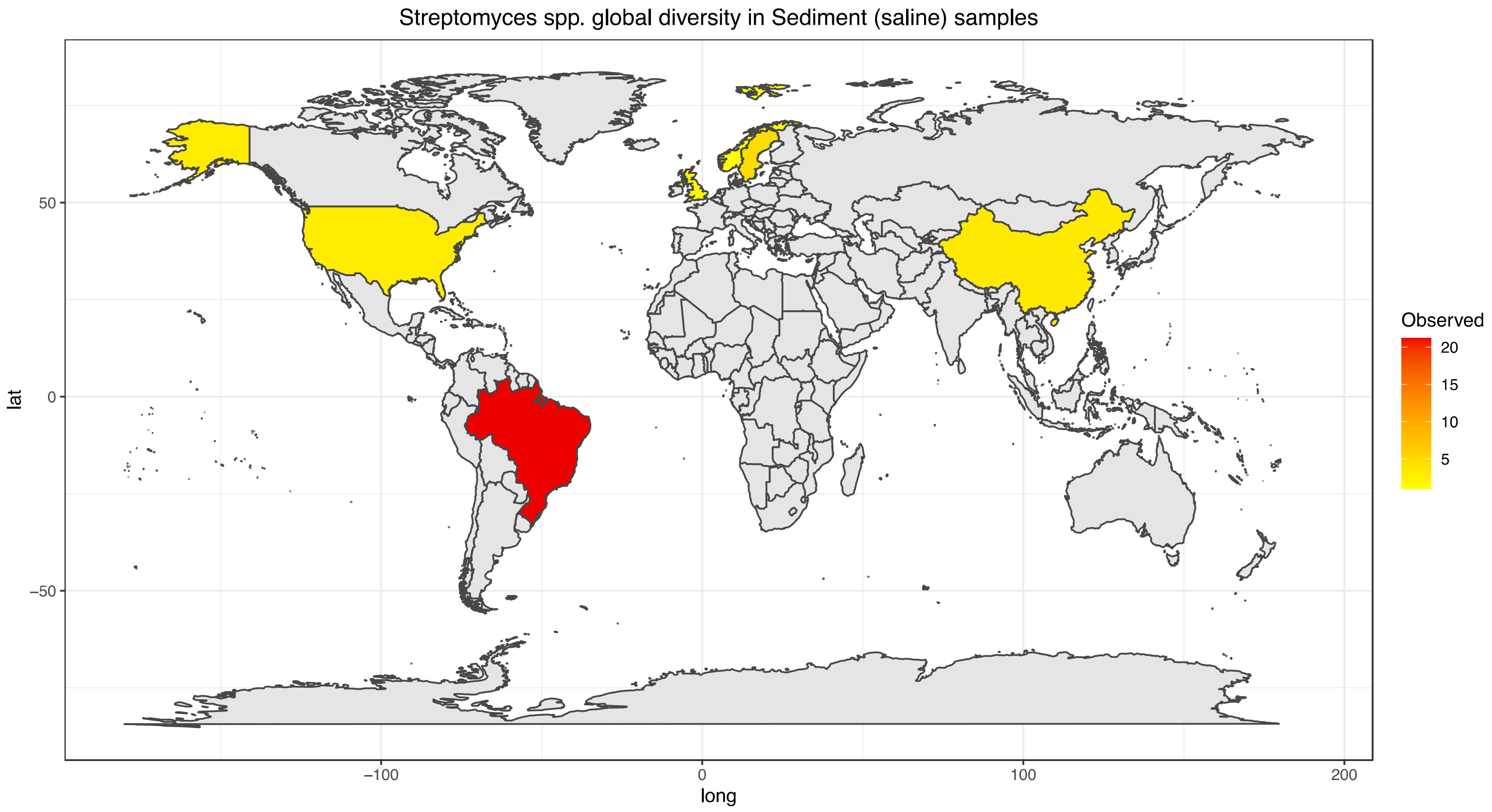

Figure S6

Figure S6. *Streptomyces* diversity map in Sediment (saline) samples showing species richness distribution worldwide. The average number of observed ASVs was shown as a gradient from lowest (yellow) to highest (red) diversity.

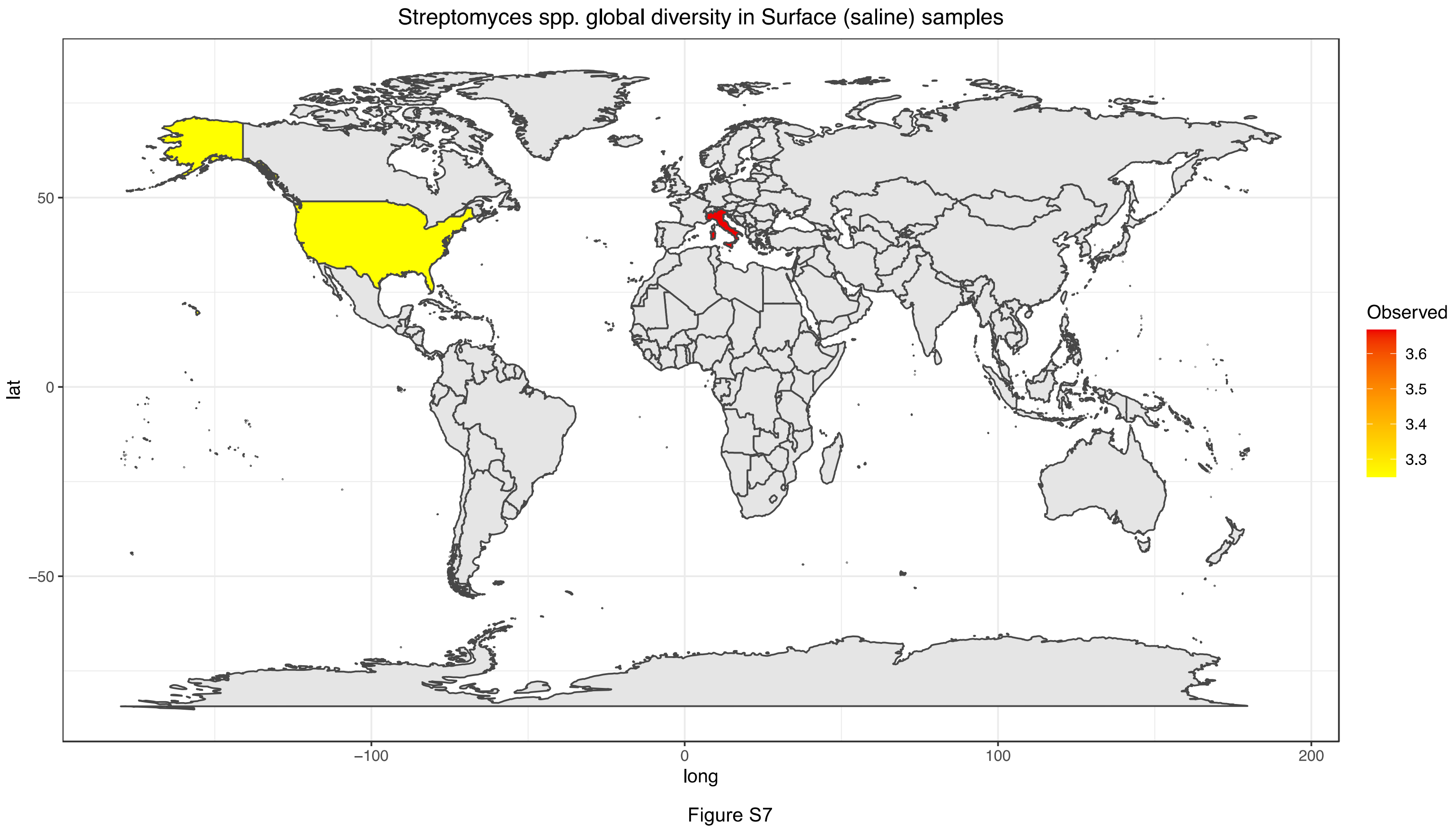

Figure S7. *Streptomyces* diversity map in Surface (saline) samples showing species richness distribution worldwide. The average number of observed ASVs was shown as a gradient from lowest (yellow) to highest (red) diversity.

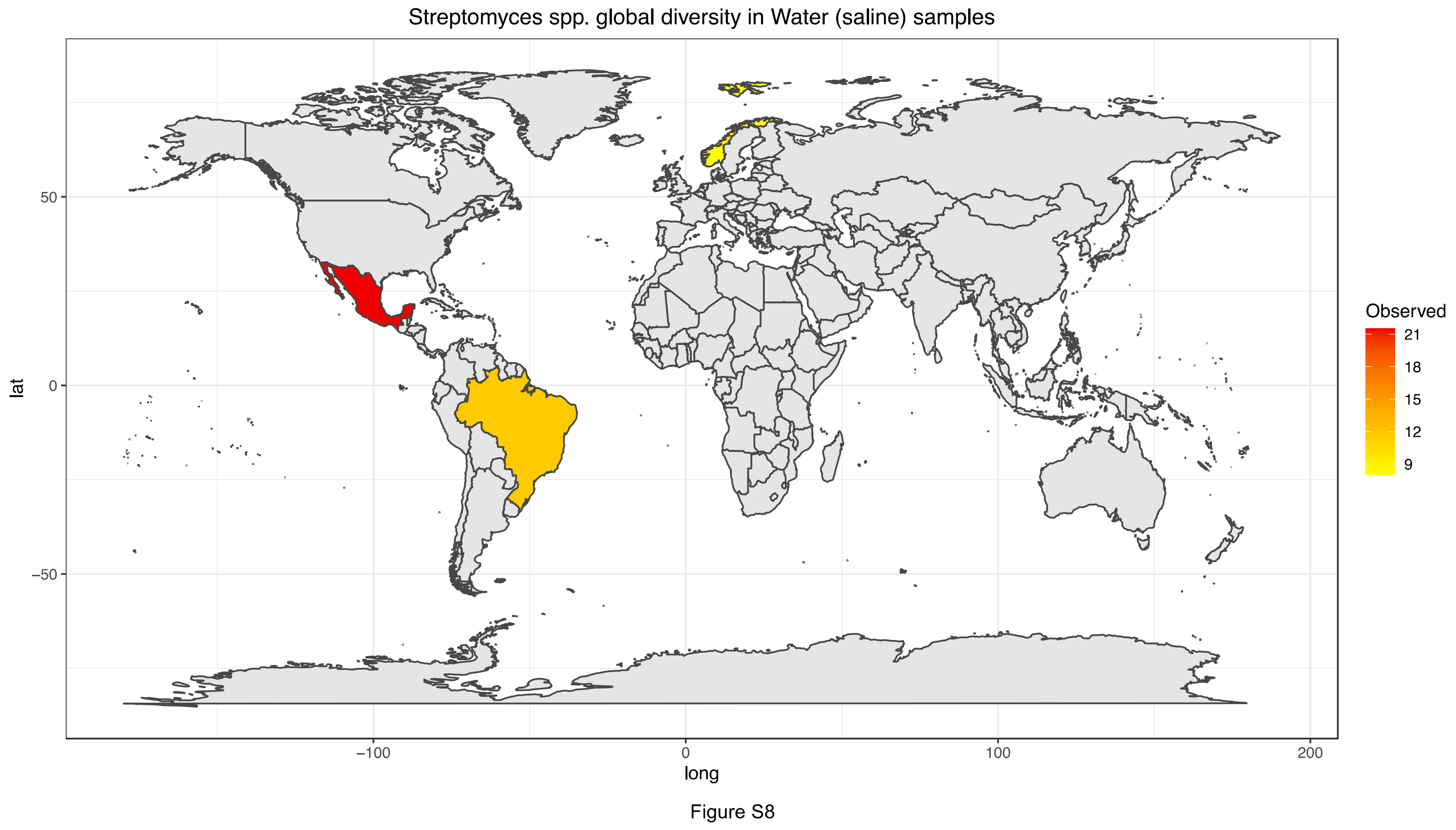

Figure S8. *Streptomyces* diversity map in Water (saline) samples showing species richness distribution worldwide. The average number of observed ASVs was shown as a gradient from lowest (yellow) to highest (red) diversity.

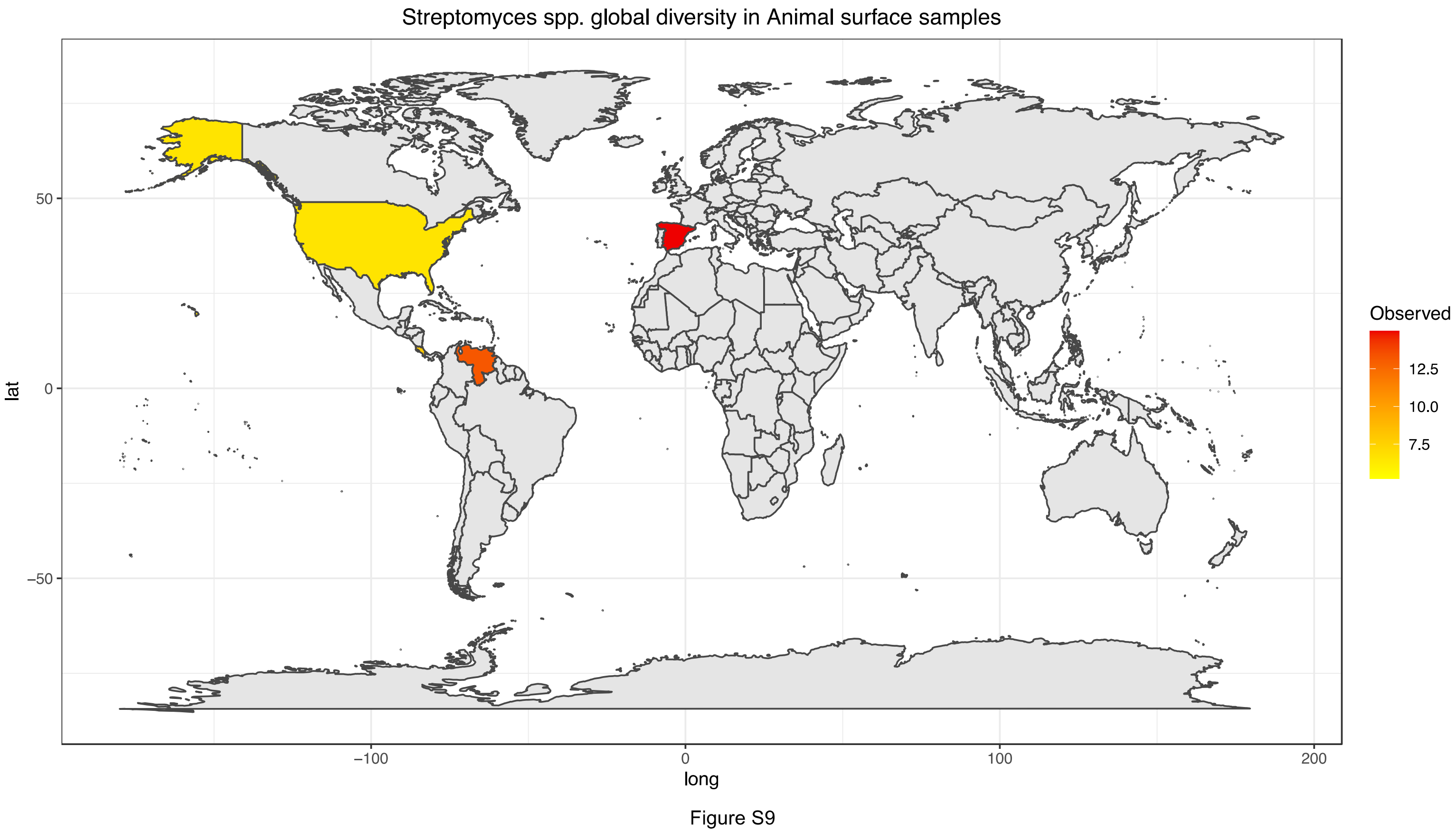

Figure S9. *Streptomyces* diversity map in Animal surface samples showing species richness distribution worldwide. The average number of observed ASVs was shown as a gradient from lowest (yellow) to highest (red) diversity.

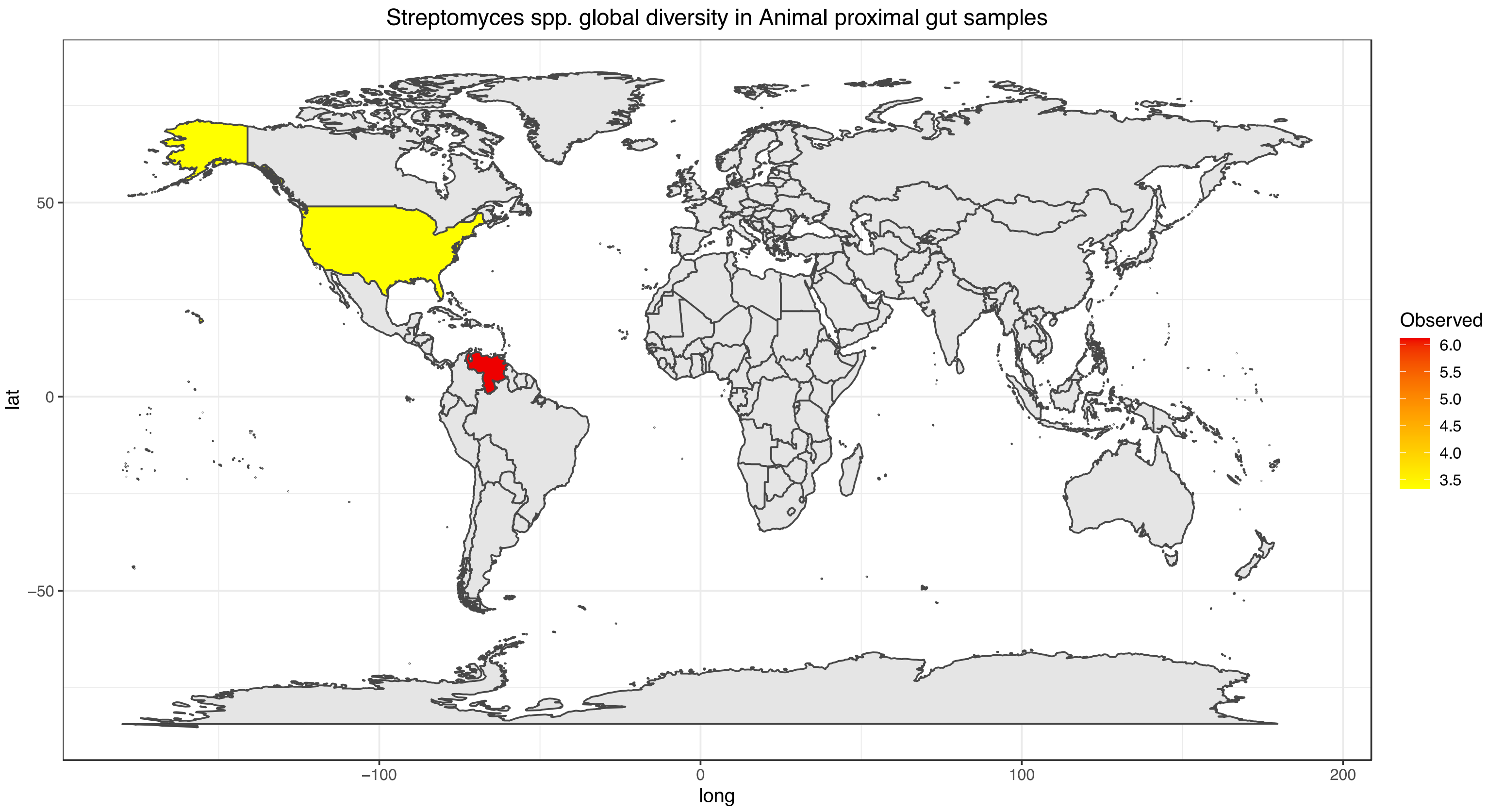

Figure S10

Figure S10. *Streptomyces* diversity map in Animal proximal samples showing species richness distribution worldwide. The average number of observed ASVs was shown as a gradient from lowest (yellow) to highest (red) diversity.

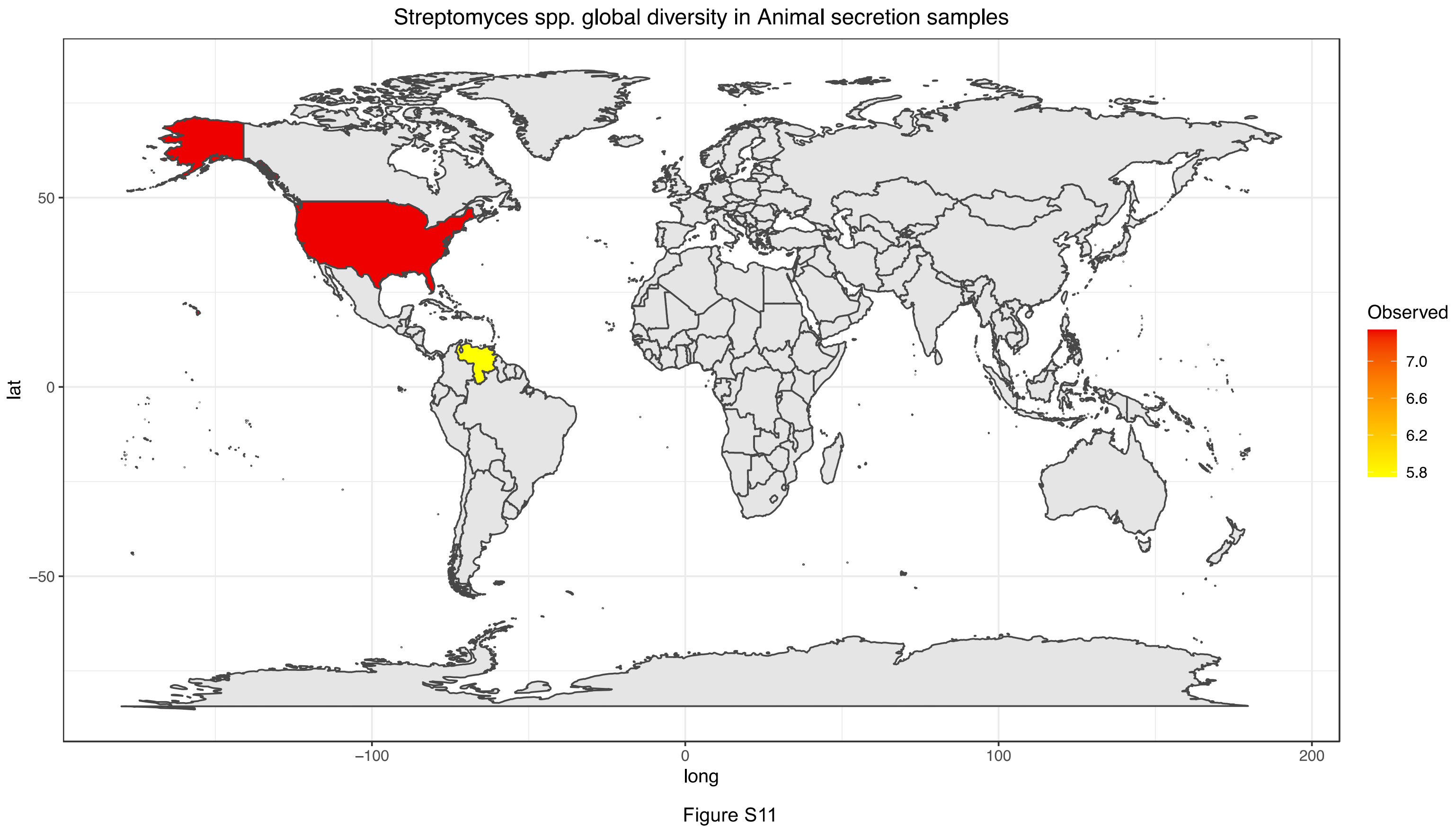

Figure S11. *Streptomyces* diversity map in Animal secretion samples showing species richness distribution worldwide. The average number of observed ASVs was shown as a gradient from lowest (yellow) to highest (red) diversity.

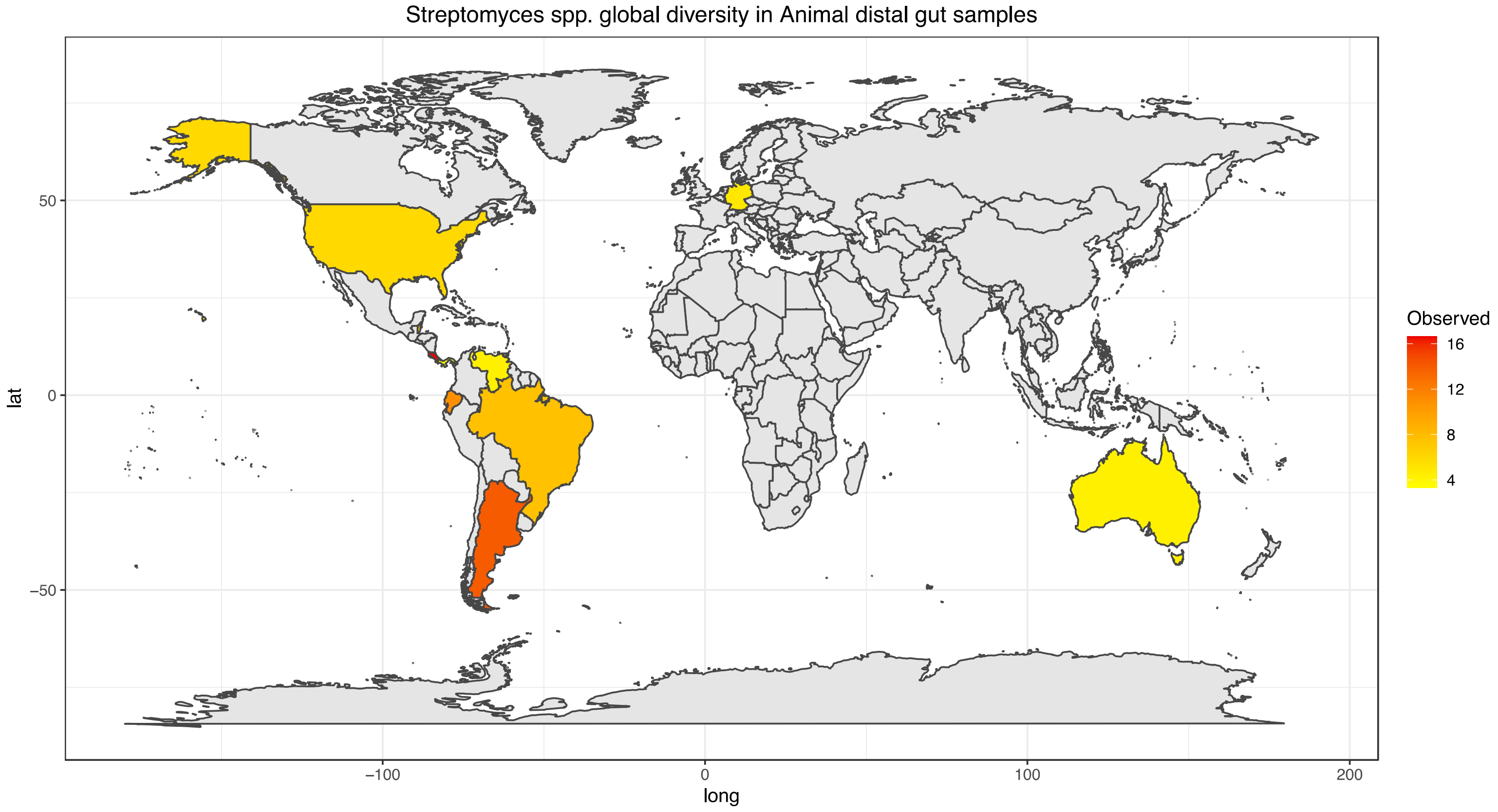

Figure S12

Figure S12. *Streptomyces* diversity map in Animal distal gut samples showing species richness distribution worldwide. The average number of observed ASVs was shown as a gradient from lowest (yellow) to highest (red) diversity.

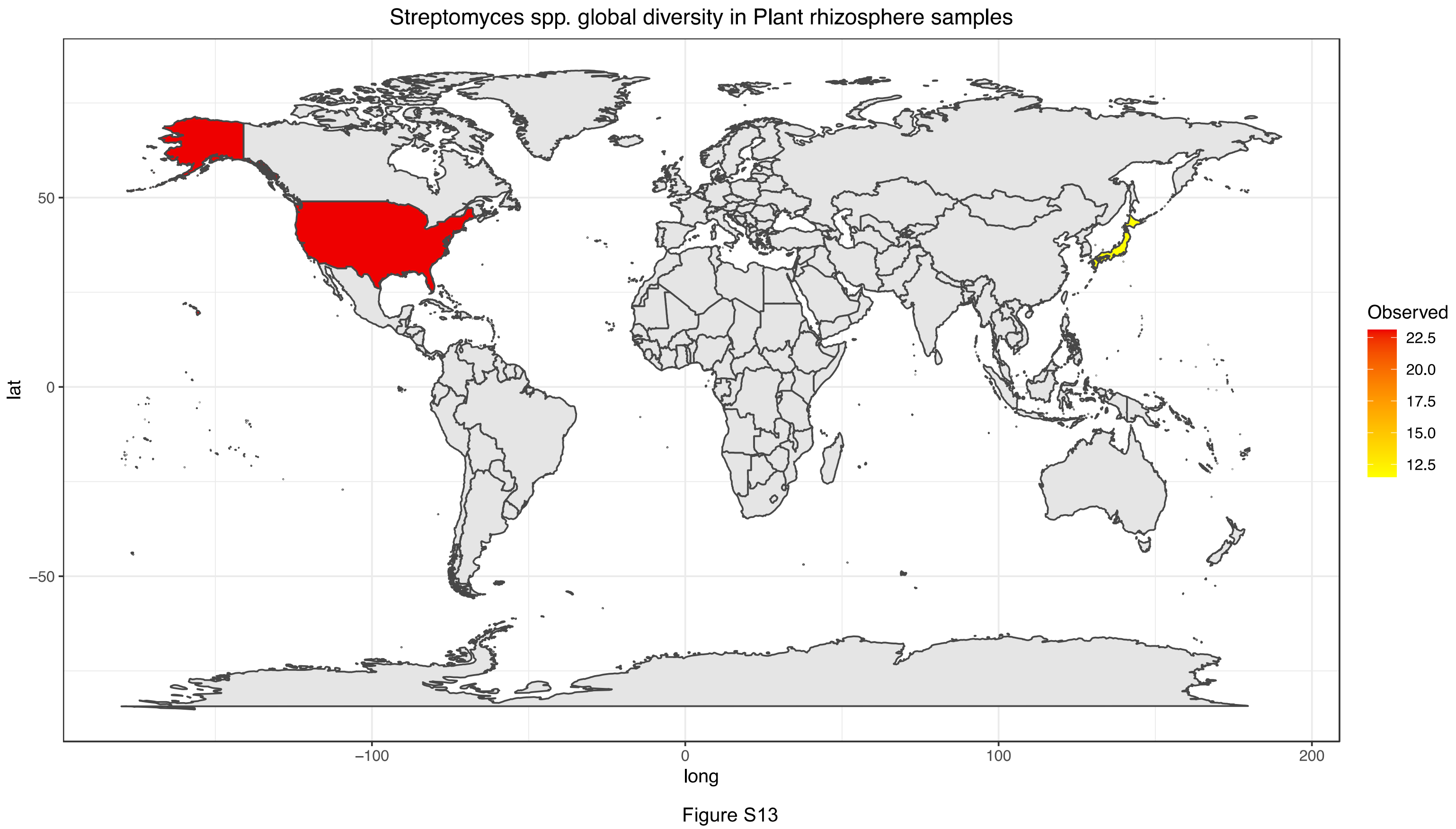

Figure S13. *Streptomyces* diversity map in Plant rhizosphere samples showing species richness distribution worldwide. The average number of observed ASVs was shown as a gradient from lowest (yellow) to highest (red) diversity.

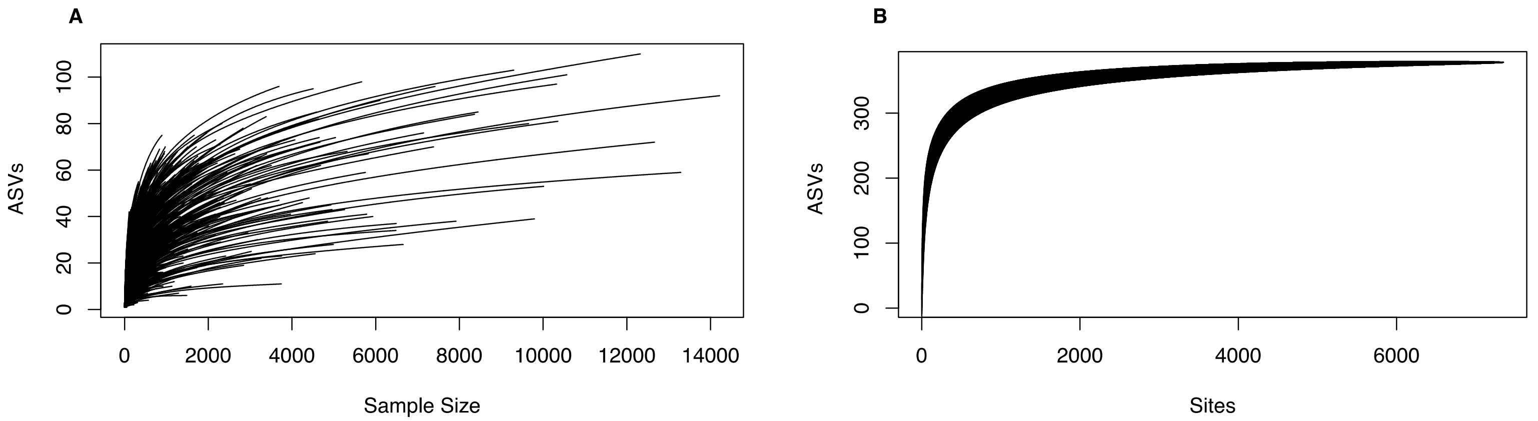

Figure S14. A) Rarefaction curve was generated using the “rarecurve” function in the R library vegan showing frequency of observed ASVs for each samples. B) Species accumulation curve was generated using “specaccum” function in the R library vegan representing the number of ASVs found at increasing number of sites(locations).
